# Provings

**Published:** 1890-07

**Authors:** E. W. Berridge


### PROVINGS.

EXTRACTED BY E. W. BERRIDGE, M. D.

*Cannabis Indica.*—The effects of *Haschisch* are given in *Cassell's Saturday Journal*, Feb. 8th, 1890, p. 476.

A lady imagined her body divided in half, the lower portion running away. Under the dreadful apprehension that life would cease if they were not quickly re-united, she gave chase to the seceding lower half.

A lady imagined her toes leaving her one by one; then her lower limbs; the fingers, forearms, arms, and lower part of the trunk followed, and just as her heart was struggling to escape she awoke. [I conclude "awoke" means "awoke from her hallucination," as there is no mention of sleep.—E. W. BERRIDGE.]

A gentleman walked ten miles, or more, visited several friends, acting rationally all the time, but without the slightest knowledge of what he had done. He was surprised on finding himself at the extreme end of the city, without knowing how he got there. When he subsequently learned of the visits he had made, carrying on conversation in a natural manner, appearing only a trifle dull, he could scarcely believe it.

*Tabacum.*—In *Titbits*, 1889, vol. 16, May, page 61, a correspondent writes that smoking caused in him color-blindness, and that on more than one occasion he gave a sovereign instead of a shilling, through this defect. This has not occurred since he ceased smoking.

